# Challenges and opportunities associated with waste management in India

**DOI:** 10.1098/rsos.160764

**Published:** 2017-03-22

**Authors:** Sunil Kumar, Stephen R. Smith, Geoff Fowler, Costas Velis, S. Jyoti Kumar, Shashi Arya, Rena, Rakesh Kumar, Christopher Cheeseman

**Affiliations:** 1 CSIR-National Environmental Engineering Research Institute (NEERI), Nehru Marg, Nagpur, India; 2 Department of Civil and Environmental Engineering, Imperial College London, London, UK; 3 School of Civil Engineering, University of Leeds, Leeds, UK; 4 Andhra Pradesh Technology Development and Promotion Centre, Confederation of Indian Industry, Hyderabad, India

**Keywords:** waste management, sustainable development, India, population growth, resource recovery, waste to energy

## Abstract

India faces major environmental challenges associated with waste generation and inadequate waste collection, transport, treatment and disposal. Current systems in India cannot cope with the volumes of waste generated by an increasing urban population, and this impacts on the environment and public health. The challenges and barriers are significant, but so are the opportunities. This paper reports on an international seminar on ‘Sustainable solid waste management for cities: opportunities in South Asian Association for Regional Cooperation (SAARC) countries’ organized by the Council of Scientific and Industrial Research-National Environmental Engineering Research Institute and the Royal Society. A priority is to move from reliance on waste dumps that offer no environmental protection, to waste management systems that retain useful resources within the economy. Waste segregation at source and use of specialized waste processing facilities to separate recyclable materials has a key role. Disposal of residual waste after extraction of material resources needs engineered landfill sites and/or investment in waste-to-energy facilities. The potential for energy generation from landfill via methane extraction or thermal treatment is a major opportunity, but a key barrier is the shortage of qualified engineers and environmental professionals with the experience to deliver improved waste management systems in India.

## 1. Introduction

Solid waste management (SWM) is a major problem for many urban local bodies (ULBs) in India, where urbanization, industrialization and economic growth have resulted in increased municipal solid waste (MSW) generation per person [1]. Effective SWM is a major challenge in cities with high population density. Achieving sustainable development within a country experiencing rapid population growth and improvements in living standards is made more difficult in India because it is a diverse country with many different religious groups, cultures and traditions.

Despite significant development in social, economic and environmental areas, SWM systems in India have remained relatively unchanged. The informal sector has a key role in extracting value from waste, with approximately 90% of residual waste currently dumped rather than properly landfilled [2]. There is an urgent need to move to more sustainable SWM, and this requires new management systems and waste management facilities. Current SWM systems are inefficient, with waste having a negative impact on public health, the environment and the economy [3]. The waste Management and Handling Rules in India were introduced by the Ministry of Environment and Forests (MoEF) [4], although compliance is variable and limited.

This paper reviews the challenges, barriers and opportunities associated with improving waste management in India. It is the output from an international seminar on ‘Sustainable solid waste management for cities: opportunities in SAARC countries' organized by the Council of Scientific and Industrial Research-National Environmental Engineering Research Institute (CSIR-NEERI) and held in Nagpur, India in 2015. SAARC is the South Asian Association for Regional Cooperation and includes Bangladesh, Bhutan, India, Maldives, Nepal, Pakistan, Sri Lanka and Afghanistan.

## 2. Waste generation in India

India is experiencing rapid urbanization while remaining a country with physical, climatic, geographical, ecological, social, cultural and linguistic diversity, as shown in table 1 [5]. The population of India was 1252 million in 2013, compared with 1028 million in 2001 [6]. Population growth is a major contributor to increasing MSW in India.

**Table 1.** Population growth in India between 1911 and 2011. Source: Provisional Population Totals-India, 2011.

census year	population × 10^6^	decadal growth ×10^6^	average annual exponential growth rate (%)	progressive growth rate compared with 1911 (%)
1911	252.0	13.7	0.56	5.75
1921	251.3	−0.8	−0.03	5.42
1931	278.9	27.6	1.04	17.02
1941	318.6	39.7	1.33	33.67
1951	361.1	42.4	1.25	51.47
1961	439.2	78.1	1.96	84.25
1971	548.1	108.9	2.20	129.94
1981	683.3	135.1	2.22	186.64
1991	846.4	163.1	2.16	255.05
2001	1028.7	182.3	1.97	331.52
2011	1210.2	181.4	1.64	407.64

### 2.1. Growth of mega cities in India

Megacities are a relatively recent phenomenon, associated with globalization of the economy, culture and technology [7]. Megacities in India include Ahmedabad (6.3 million), Hyderabad (7.7 million), Bangalore (8.4 million), Chennai (8.6 million), Kolkata (14.1 million), Delhi (16.3 million) and Greater Mumbai (18.4 million [6]). These have dynamic economic growth and high waste generation *per capita*, as shown in table 2 [7].

**Table 2.** Major cities in India and *per capita* waste generation data (2010–2011). Source: *Census of India 2011, ^#^CPCB Report 2011.

city	*population (2011) × 10^6^	^#^total waste generated in tonnes per day	waste generation (kg *per capita* per day)
Ahmedabad	6.3	2300	0.36
Hyderabad	7.7	4200	0.54
Bangalore	8.4	3700	0.44
Chennai	8.6	4500	0.52
Kolkata	14.1	3670	0.26
Delhi	16.3	5800	0.41
Mumbai	18.4	6500	0.35

### 2.2. Infrastructure development for public health and protection of the environment

Improvements in civil infrastructure are required for India to become a world leading economy. Developing high-quality infrastructure that meets the needs of the people and protects the environment is fundamental to achieving effective economic growth [8]. Waste management infrastructure has an important role in delivering sustainable development. Rapid population growth in India has led to depletion of natural resources. Wastes are potential resources and effective waste management with resource extraction is fundamental to effective SWM. Value extraction from waste can be materials, energy or nutrients, and this can provide a livelihood for many people [7]. The transition from wastes to resources can only be achieved through investment in SWM as this depends on a coordinated set of actions to develop markets and maximize recovery of reusable/recyclable materials [9]. Materials, energy and nutrient recovery must be the aim of future SWM infrastructure development in India. Resources can be recovered from wastes using existing technologies and India has an extremely effective recycling tradition. The ‘scrap dealer’ systems produce recycled materials through an extensive and well-coordinated network across the country.

### 2.3. Statistics on waste generation and waste characterization data

Estimating the quantity and characteristics of MSW in India and forecasting future waste generation is fundamental to successful waste management planning [10]. The quantity of MSW generated depends on living standards, the extent and type of commercial activity, eating habits and season [11]. India generates approximately 133 760 tonnes of MSW per day, of which approximately 91 152 tonnes is collected and approximately 25 884 tonnes is treated [12]. MSW generation *per capita* in India ranges from approximately 0.17 kg per person per day in small towns to approximately 0.62 kg per person per day in cities, as shown in table 3 [13].

**Table 3.** Waste generation *per capita* in Indian cities. Source: Kumar *et al*. [13,14].

population	waste generation rate (kg *per capita* per day)
cities with a population <0.1 million (eight cities)	0.17–0.54
cities with a population of 0.1–0.5 million (11 cities)	0.22–0.59
Cities with a population 1–2 million (16 cities)	0.19–0.53
Cities with a population >2 million (13 cities)	0.22–0.62

**Figure 1.**
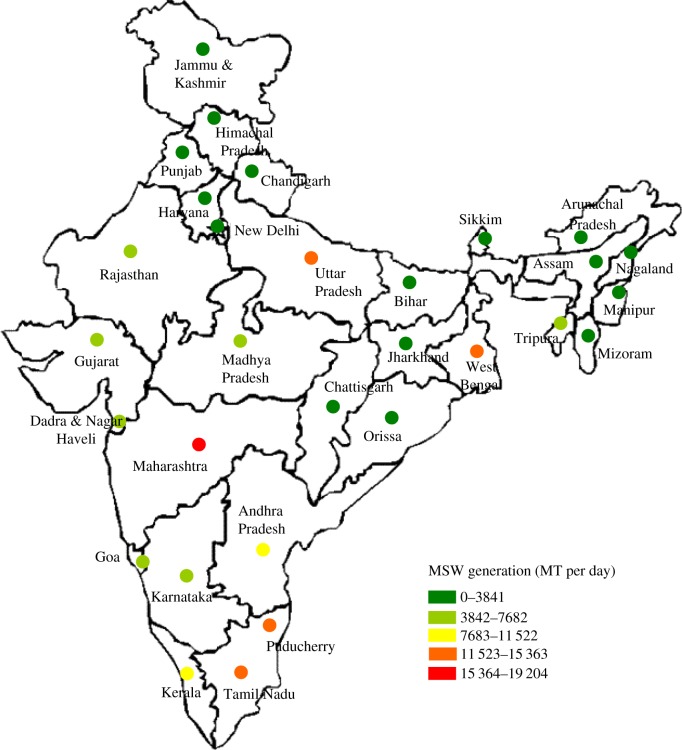
State-level statistics of MSW generation in India (2009–2012). Source: Central Pollution Control Board, Govt. of India, 2012.

Waste generation rate depends on factors such as population density, economic status, level of commercial activity, culture and city/region. Figure 1 provides data on MSW generation in different states, indicating high waste generation in Maharashtra (115 364–19 204 tonnes per day), Uttar Pradesh, Tamil Nadu, West Bengal (11 523–15 363 tonnes per day), Andhra Pradesh, Kerala (7683–11 522 tonnes per day) and Madhya Pradesh, Rajasthan, Gujarat, Karnataka and Mizoram (3842–7662 tonnes per day). Lower waste generation occurs in Jammu and Kashmir, Bihar, Jharkhand, Chhattisgarh, Orissa, Goa, Assam, Arunachal Pradesh, Meghalaya, Tripura, Nagaland and Manipur (less than 3841 tonnes per day).

### 2.4. Waste characterization data

The local economy impacts on waste composition, as high-income groups use more packaged products, resulting in higher volumes of plastics, paper, glass, metals and textiles. Changes in waste composition can have a significant impact on waste management practices [9]. MSW may also contain hazardous wastes such as pesticides, paints, used medicine and batteries. Compostable organics include fruits, vegetables and food waste. Healthcare waste contains disposable syringes, sanitary materials and blood containing textiles and is governed by the Biomedical Waste (Management and Handling) Rules 1998 and the Amended Rules, 2003, and should not be mixed with MSW [5,15]. The average composition of MSW produced by Indian cities is approximately 41 wt.% organic, approximately 40 wt.% inert, with approximately 19 wt.% potentially recyclable materials, as shown in table 4 [16]. Most organic waste is generated from households, and inert waste is generated from construction, demolition and road sweeping. Waste samples collected from Delhi, Ahmadabad and Bangalore indicate that MSW composition varies between cities [14,17].

**Table 4.** Average (% by weight) composition of MSW in Indian metro cities. Source: Sharholy *et al*. [16].

percentage (%) by weight							
compostable	inert	paper	plastic	glass	metals	textile	leather
41	40	6	4	2	2	4	1

### 2.5. Predictions on future waste growth

World waste production is expected to be approximately 27 billion tonnes per year by 2050, one-third of which will come from Asia, with major contributions from China and India [18]. Waste generation in urban areas of India will be 0.7 kg per person per day in 2025, approximately four to six times higher than in 1999. The problems associated with waste become more acute as the size of communities increase and this provides opportunities for decentralized waste management by self-help groups and NGOs [19]. The waste produced in urban areas of India is approximately 170 000 tonnes per day, equivalent to about 62 million tonnes per year, and this is expected to increase by 5% per year owing to increases in population and changing lifestyles [20]. Table 5 shows that urban India generated 31.6 million tonnes of waste in 2001 and is currently generating 47.3 million tonnes. By 2041, waste generation is predicted to be 161 million tonnes, a fivefold increase in four decades [21].

**Table 5.** Predicted population growth and overall impact on waste generation. Source: Amepu [21].

year	population (×10^6^)	*per capita* generation (kg per day)	total waste generation (x 10^3^ Tonnes per year)
2001	197.3	0.439	31.63
2011	260.1	0.498	47.30
2021	342.8	0.569	71.15
2031	451.8	0.649	107.01
2036	518.6	0.693	131.24
2041	595.4	0.741	160.96

## 3. Current waste management in India

### 3.1. Key waste management legislations in India

The MoEF issued MSW (Management and Handling) Rules 2000 to ensure proper waste management in India and new updated draft rules have recently been published [4]. Municipal authorities are responsible for implementing these rules and developing infrastructure for collection, storage, segregation, transportation, processing and disposal of MSW. Chandigarh is the first city to develop SWM in a planned way and has improved waste management compared with other Indian cities [22].

### 3.2. Role of the informal sector in waste materials reuse and recycling

The informal sector has a very important role in India and this must be integrated into formal SWM systems [15,21]. The informal sector is characterized by small-scale, labour-intensive, largely unregulated and unregistered low-technology manufacturing or provision of materials and services [23]. Waste pickers collect household or commercial/industrial waste and many hundreds of thousands of waste pickers in India depend on waste for an income, despite the associated health and social issues. Pickers extract potential value from waste bins, trucks, streets, waterways and dumpsites. Some work in recycling plants owned by cooperatives or waste picker associations. Waste picking is often the only source of income for families, providing a livelihood for significant numbers of urban poor and usable materials to other enterprises. Waste pickers in Pune collect organic waste for composting and biogas generation. Waste pickers also make a significant contribution by keeping cities clean.

A recent study of six Indian cities found that waste pickers recovered approximately 20% of waste, with 80 000 people involved in recycling approximately three million tonnes. It is estimated that every tonne of recyclable material collected saved the ULB approximately INR 24 500 per annum and avoided the emission of 721 kg CO_2_ per annum [21].

### 3.3. Waste collection and transport

Waste collection, storage and transport are essential elements of any SWM system and can be major challenges in cities. Waste collection is the responsibility of the municipal corporations in India, and bins are normally provided for biodegradable and inert waste [24–26]. Mixed biodegradable and inert waste is often dumped, with open burning a common practice. Improvements to waste collection and transport infrastructure in India will create jobs, improve public health and increase tourism [27]. Local bodies spend around Rs. 500–1000 per tonne on SWM with 70% of this amount spent on collection and 20% spent on transport.

### 3.4. Waste disposal

**Figure 2.**
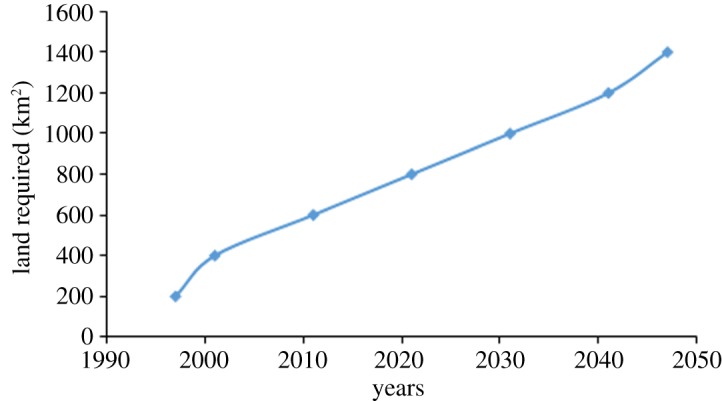
Cumulative land required (km^2^) for disposal of MSW. Source: Singhal & Pandey [29].

SWM disposal is at a critical stage of development in India. There is a need to develop facilities to treat and dispose of increasing amounts of MSW [28]. More than 90% of waste in India is believed to be dumped in an unsatisfactory manner. It is estimated that approximately 1400 km^2^ was occupied by waste dumps in 1997 and this is expected to increase in the future, as shown in figure 2 [29,30].

Properly engineered waste disposal protects public health and preserves key environmental resources such as ground water, surface water, soil fertility and air quality. Indian cities with containment landfill sites include Mumbai, Kolkata, Chennai, Nashik, Vadodara, Jamshedpur, Allahabad, Amritsar, Rajkot, Shimla, Thiruvananthapuram and Dehradun [13]. Table 6 shows treatment facilities available in different states in India and table 7 has information on landfills associated with different cities.

**Table 6.** State-wise^a^ status of MSW processing facilities in India in 2011. Source: Planning Commission 2014 [20].

state	composting	vermicomposting	biomethanation	pelletization	waste to energy
Andaman and Nicobar	1	nil	nil	nil	nil
Andhra Pradesh	24	nil	nil	11	2
Assam	1	nil	nil	nil	nil
Chandigarh	Nil	nil	nil	1	nil
Chattisgarh	6	nil	nil	nil	nil
Delhi	3	nil	nil	nil	3
Goa	14	nil	nil	nil	nil
Gujarat	3	93	nil	6	nil
Himachal Pradesh	10	nil	nil	nil	nil
Jammu and Kashmir	1	nil	nil	nil	nil
Jharkhand	4	nil	nil	nil	nil
Kerala	21	7	10	1	1
Madhya Pradesh	7	nil	nil	2	nil
Maharashtra	6	2	5	5	2
Meghalaya	1	1	nil	nil	nil
Nagaland	1	1	nil	nil	nil
Orissa	1	nil	nil	nil	nil
Punjab	1	3	nil	nil	nil
Sikkim	1	nil	nil	nil	nil
Tamil Nadu	162	24	nil	3	nil
Tripura	1	nil	nil	nil	nil
West Bengal	13	7	nil	nil	nil
total	279	138	172	29	8

^a^All other states and UTs currently have no processing facilities.

**Table 7.** Landfill sites associated with different cities in India. Source: Parvathamma [31].

city	number of landfills	area of landfills (hectare)
Chennai	2	465.5
Coimbatore	2	292
Surat	1	200
Greater Mumbai	3	140
Greater Hyderabad	1	121.5
Ahmadabad	1	84
Delhi	3	66.4
Jabalpur	1	60.7
Indore	1	59.5
Madurai	1	48.6
Greater Bangalore	2	40.7
Greater Vishakhapatnam	1	40.5
Ludhiana	1	40.4
Nasik	1	34.4
Jaipur	3	31.4
Srinagar	1	30.4
Kanpur	1	27
Kolkata	1	24.7
Chandigarh	1	18
Ranchi	1	15
Raipur	1	14.6
Meerut	2	14.2
Guwahati	1	13.2
Thiruvananthapuram	1	12.5

### 3.5. Environmental and health impacts of waste dumping

Waste dumps have adverse impacts on the environment and public health [32–37]. Open dumps release methane from decomposition of biodegradable waste under anaerobic conditions. Methane causes fires and explosions and is a major contributor to global warming [9]. There are also problems associated with odour and migration of leachates to receiving waters [38]. Odour is a serious problem, particularly during the summer when average temperatures in India can exceed 45°C [39]. Discarded tyres at dumps collect water, allowing mosquitoes to breed, increasing the risk of diseases such as malaria, dengue and West Nile fever. Uncontrolled burning of waste at dump sites releases fine particles which are a major cause of respiratory disease and cause smog [9]. Open burning of MSW and tyres emits 22 000 tonnes of pollutants into the atmosphere around Mumbai every year [21]. The impacts of poor waste management on public health are well documented, with increased incidences of nose and throat infections, breathing difficulties, inflammation, bacterial infections, anaemia, reduced immunity, allergies, asthma and other infections [40].

## 4. Engineered landfills in India

The UN Environmental Programme defines landfill as the controlled disposal of MSW on land in such a way that contact between waste and the environment is significantly reduced, with waste disposal concentrated in a well-defined area. Engineered landfill allows the safe disposal of residual MSW on land, but protects ground and surface water from pollution and avoids air emissions, wind-blown litter, odour, fire hazards, problems with animals, birds and other pests/rodents, and reduces greenhouse gas (GHG) emissions and slope instability issues [4]. Properly managed engineered landfills should replace dumps in India. This would significantly reduce the environmental impact of waste [41].

## 5. Waste-to-energy in India

The problems associated with improper waste disposal could be significantly mitigated by requiring material recovery. Source separation of inert and high moisture content fractions would maximize the potential for thermal recovery and other treatment options in India. The waste processed in thermal recovery is residual waste that remains after all commercially viable recyclable materials have been extracted. Waste-to-energy technologies produce energy, recover materials and free land that would otherwise be used for dumping. The composition of residual waste is important for energy recovery and waste composition is changing in India, with the amount of high calorific waste generally increasing [42]. A significant increase in the use of waste-to-energy technologies has been proposed, but this depends on location, climate, demographics and other socioeconomic factors [20,38,43].

The most widely used waste-to-energy technology for residual waste uses combustion to provide combined heat and power [44]. Adopting maximum recycling with waste-to-energy in an integrated waste management system would significantly reduce dumping in India. Waste-to-energy technologies are available that can process unsegregated low-calorific value waste, and industry is keen to exploit these technologies in India. Several waste-to-energy projects using combustion of un-segregated low-calorific value waste are currently being developed. Alternative thermal treatment processes to combustion include gasification, pyrolysis, production of refuse derived fuel and gas-plasma technology.

Waste-to-energy development in India is based on a build, operate and transfer model. Increased waste-to-energy would reduce disposal to land and generate clean, reliable energy from a renewable fuel source, reducing dependence on fossil fuels and reducing GHG emissions. In addition, generation of energy from waste would have significant social and economic benefits for India. However, the track record of waste-to-energy in India highlights some of the difficulties. The vast majority of facilities have not worked effectively due to various operational and design problems. For example, the first large-scale MSW incinerator built at Timarpur, New Delhi in 1987 had a capacity to process 300 tonnes per day and cost Rs. 250 million (US$ 5.7 million). The plant failed because of poor waste segregation, seasonal variations in waste composition and properties, inappropriate technology selection and operational and maintenance issues [45]. Despite this experience, waste-to-energy will have a key role in future waste management in India.

## 6. Barriers to improved waste management in India

The current status of SWM in India is poor because the best and most appropriate methods from waste collection to disposal are not being used. There is a lack of training in SWM and the availability of qualified waste management professionals is limited. There is also a lack of accountability in current SWM systems throughout India [46]. Municipal authorities are responsible for managing MSW in India but have budgets that are insufficient to cover the costs associated with developing proper waste collection, storage, treatment and disposal. The lack of strategic MSW plans, waste collection/segregation and a government finance regulatory framework are major barriers to achieving effective SWM in India.

Limited environmental awareness combined with low motivation has inhibited innovation and the adoption of new technologies that could transform waste management in India. Public attitudes to waste are also a major barrier to improving SWM in India.

## 7. Changes required to improve waste management in India

Core to the vision for waste management in India is the use of wastes as resources with increased value extraction, recycling, recovery and reuse. ULBs need to be responsible for waste management, with the ULB Commissioner and Chairman directly responsible for performance of waste management systems. Waste management needs to be regarded throughout Indian society as an essential service requiring sustainable financing. The case presented to a ULB for a properly funded system must demonstrate the advantages of sound investment in waste management.

A strong and independent authority is needed to regulate waste management if SWM is to improve in India. Without clear regulation and enforcement, improvements will not happen. Strong waste regulations can drive innovation. The waste management sector needs to include attractive and profitable businesses with clear performance requirements imposed by the ULB, with financial penalties applied when waste management services are not working effectively. Finance for waste management companies and funding for infrastructure must be raised from waste producers through a waste tax. An average charge of 1 rupee per person per day would generate close to 50 000 crores annually, and this level of funding would probably be sufficient to provide effective waste management throughout India.

Information on future quantities and characterization of wastes is essential as this determines the appropriateness of different waste management and treatment options. State-level procurement of equipment and vehicles is necessary for primary and secondary collection with effective systems for monitoring collection, transport and disposal.

Littering and waste in streets is a major problem in India that has serious impacts on public health. Nagpur has introduced a system for sweeping roads in which every employee sweeps a fixed road length. The Swatchata Doot Aplya Dari (sanitary worker at your doorstep) scheme of the Centre for Development Communication was selected as an example of good practice by UN HABITAT in 2007.

Waste management must involve waste segregation at source to allow much more efficient value extraction and recycling. Separating dry (inorganic) and wet (biodegradable) waste would have significant benefits and should be the responsibility of the waste producer.

Long-term waste management planning requires visionary project development by ULBs, the private sector and NGOs. The roles and responsibilities to deliver sustainable systems need to be defined, with monitoring and evaluation to monitor progress. Experiences should be shared between different regions of India and different social groups. There are a number of research institutes, organizations, NGOs and private sector companies working on a holistic approach to SWM, and future waste management in India must involve extensive involvement of the informal sector throughout the system.

There is a need to develop training and capacity building at every level. All Indian school children should understand the importance of waste management, the effects of poor waste management on the environment and public health, and the role and responsibilities of each individual in the waste management system. This will develop responsible citizens who regard waste as a resource opportunity.

## 8. Conclusion

Population growth and particularly the development of megacities is making SWM in India a major problem. The current situation is that India relies on inadequate waste infrastructure, the informal sector and waste dumping. There are major issues associated with public participation in waste management and there is generally a lack of responsibility towards waste in the community. There is a need to cultivate community awareness and change the attitude of people towards waste, as this is fundamental to developing proper and sustainable waste management systems. Sustainable and economically viable waste management must ensure maximum resource extraction from waste, combined with safe disposal of residual waste through the development of engineered landfill and waste-to-energy facilities. India faces challenges related to waste policy, waste technology selection and the availability of appropriately trained people in the waste management sector. Until these fundamental requirements are met, India will continue to suffer from poor waste management and the associated impacts on public health and the environment.

## Data accessibility

No data were generated from the work, and all supporting data were obtained from previously published work available via the references below and from the output of the international seminar on which the paper is based.
